# Biological Actions of the Hsp90-binding Immunophilins FKBP51 and FKBP52

**DOI:** 10.3390/biom9020052

**Published:** 2019-02-01

**Authors:** Nadia R. Zgajnar, Sonia A. De Leo, Cecilia M. Lotufo, Alejandra G. Erlejman, Graciela Piwien-Pilipuk, Mario D. Galigniana

**Affiliations:** 1 Instituto de Biología y Medicina Experimental/CONICET, Buenos Aires 1428, Argentina; nadiazgajnar@gmail.com (N.R.Z.); cecilialotufo@yahoo.com.ar (C.M.L.); gpiwien@conicet.gov.ar (G.P.-P.); 2 Departamento de Química Biológica, Facultad de Ciencias Exactas y Naturales, Universidad de Buenos Aires-CONICET, Buenos Aires 1428, Argentina; sonydeleo@hotmail.com (S.D.L.); erlejman@qb.fcen.uba.ar (A.G.E.)

**Keywords:** FKBP51, FKBP52, Hsp90, dynein, telomerase, NF-κB, neurodifferentiation, cell differentiation

## Abstract

Immunophilins are a family of proteins whose signature domain is the peptidylprolyl-isomerase domain. High molecular weight immunophilins are characterized by the additional presence of tetratricopeptide-repeats (TPR) through which they bind to the 90-kDa heat-shock protein (Hsp90), and via this chaperone, immunophilins contribute to the regulation of the biological functions of several client-proteins. Among these Hsp90-binding immunophilins, there are two highly homologous members named FKBP51 and FKBP52 (FK506-binding protein of 51-kDa and 52-kDa, respectively) that were first characterized as components of the Hsp90-based heterocomplex associated to steroid receptors. Afterwards, they emerged as likely contributors to a variety of other hormone-dependent diseases, stress-related pathologies, psychiatric disorders, cancer, and other syndromes characterized by misfolded proteins. The differential biological actions of these immunophilins have been assigned to the structurally similar, but functionally divergent enzymatic domain. Nonetheless, they also require the complementary input of the TPR domain, most likely due to their dependence with the association to Hsp90 as a functional unit. FKBP51 and FKBP52 regulate a variety of biological processes such as steroid receptor action, transcriptional activity, protein conformation, protein trafficking, cell differentiation, apoptosis, cancer progression, telomerase activity, cytoskeleton architecture, etc. In this article we discuss the biology of these events and some mechanistic aspects.

## 1. Introduction

**Figure 1:**
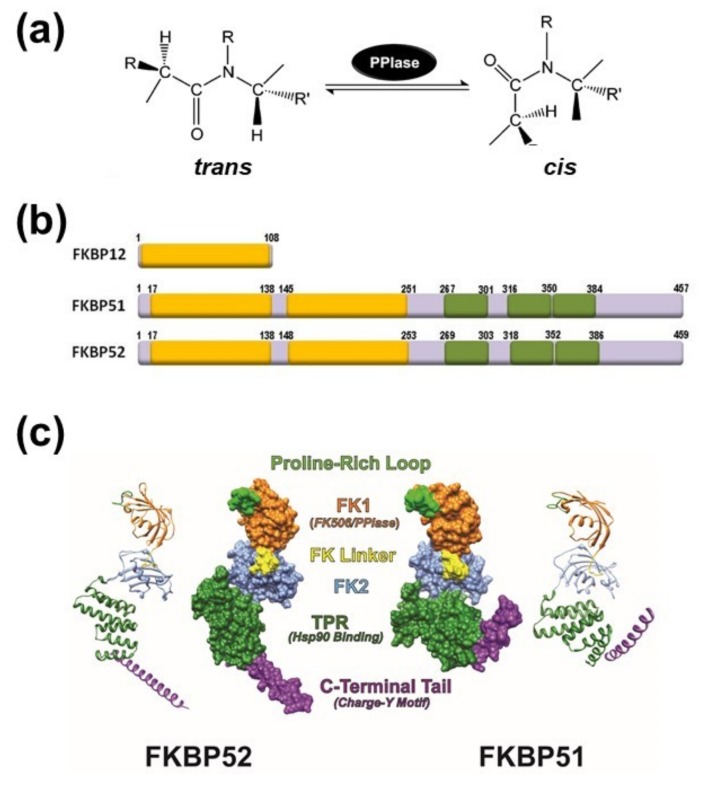
(a) Schematic representation of the peptidyl-prolyl isomerase (*cis/trans*) isomerization activity (PPIase). (b) Schematic structures of FK506-binding protein of 12-kDa (FKBP12) (acc.# AAA58476), FKBP51 (acc.# Q13451) and FKBP52 (acc.# NP_002005). The PPIase domain is depicted as yellow boxes. Only the FK1 domain (the extreme N-terminal of the protein) has PPIase activity. Tetratricopeptide repeats (TPRs) are shown in green color. (c) Ribbon and molecular surface depictions of hFKBP51 crystallo-graphic structure (right) and overlapping fragments encompassing the full length hFKBP52 crystallographic structure (left) are also shown. Note in both proteins that the FK1 domains (orange) containing the PPIase catalytic pocket and the Proline-Rich Loop (green) are connected to the FK2 domain (blue) by the FK Linker (yellow). Both proteins show TPR domains (purple) where Hsp90 binds. Structures were derived from the RCSB PDB (FKBP51: 1KT0; FKBP52: 1Q1C & 1P5Q) with Viewer-Lite 5.0 (Sharpened Productions Inc, Sioux City, IA, USA).

Immunophilins comprise a family of proteins that show two main features: a) they have a specific sequence that usually has peptidyl-prolyl-(*cis/trans*)-isomerase (PPIase) activity, i.e., the reversible *cis/trans* interconversion of Xaa-Pro bonds (see Figure 1a); b) they also have the capability to bind immunosuppressive drugs to the same PPIase domain. The classic binding ligands are FK506 (tacrolimus), rapamycin (sirolimus) or cyclosporine A, and all these drug-protein interactions abolish the PPIase enzymatic activity when the isomerase function is present in the protein. Regardless of these two conventional properties, the common feature of the family is the existence of a relatively conserved sequence in most of the members, the PPIase domain, which represents the signature domain of the entire family. Most researchers in the field often indistinctly use either term (immunophilin or PPIase protein) since they were simultaneously originated during the early times when these proteins were discovered and characterized by the binding capacity for immunosuppressive drugs and the enzymatic activity of protein *cis/trans* isomerase. For human multidomain FKBPs (FK506-binding proteins) such as FKBP25, FKBP51, FKBP52, and FKBP65, good catalysis of the *cis/trans* isomerization of the peptidyl prolyl bond using oligopeptide substrates has already been demonstrated [1]. However, despite possessing a PPIase domain not all members show significant isomerase enzymatic activity (e.g. FKBP38, FKBPL, etc.) or it is negligible or absent (e.g. FKBP37, PP5, etc.). For the case of the cyclophilin subfamily (CyP), no PPIase activity has been demonstrated to date for some members such as CyP35, CyP54, CyP57 and CyP60.

Based on the property to recognize different ligands, PPIases (EC 5.2.1.8) were classically grouped into two subfamilies [2]. They are named cyclophilins (or CyPs) when they bind cyclosporine A, or FKBPs when they bind FK506. Both drugs are not chemically-related. FK506 is a natural compound belonging to the family of macrolides that was first isolated from the bacterium *Streptomyces tsukubaensis* [3,4], whereas cyclosporine A is a cyclic undecapeptide first isolated from the fungus *Trichoderma polysporum* [5] that contains a single D-amino acid rarely encountered in nature. Unlike most peptides, cyclosporine A is not synthesized by ribosomes [6]. 

In addition to these two subfamilies, a third subfamily clusters a few numbers of proteins with PPIase activity that show homology with bacterial Parvulin. In eukaryotes, the Parvulin subfamily comprises three main members [see recent revisions in [7,8]—Pin1 (a cell cycle regulator with important roles during the transition from G2 to M phase and folding of several key proteins such as the amyloid), Par14 and Par17 (an N-terminal modified variety of Par14) that regulate the progression of the cell-cycle as well as ribosome biogenesis and several metabolic pathways. Parvulins conserve the homology in the PPIase domain, but they show no capacity to bind classic immunosuppressive drugs. 

Recently, a new type of immunophilin that represents two types of proteins has been discovered in monocellular organisms: the FCBP/CFBP (FK506- and cyclosporine-binding protein/Cyclosporine- and FK506-binding protein) family. They represent a naturally occurring chimera of both types of immunophilins, FKBPs and CyPs, connected by a flexible linker peptide, and can recognize both types of drugs, FK506 and cyclosporine A. The module sequences are: [CyP]-[linker domain]-[FKBP] for CFBP, and [FKBP]-[3TPR]-[CyP] for FCBP. They also exhibit distinct organism preference, the CFBP being found in prokaryotes, and the FCBP in eukaryotes [9]. 

## 2. The PPIase Activity Affects Protein Conformation

During the translational process in the eukaryotic ribosome, the nascent polypeptide that emerges vectorially from the exit tunnel of the large subunit is rapidly folded in a process known as “co-translational protein folding” [10]. The nascent peptide normally acquires its most stable conformation from the thermodynamic point of view, i.e. the *trans*-conformation. Because there is a relatively high percentage of the nascent polypeptides whose structures show peptidyl-prolyl bonds (i.e., 5-7% of Xaa-Pro bonds), they are able to exchange its structure from *trans-* to *cis*-conformation in subsequent maturation steps of the protein folding process, and/or assembly with other proteins in hetero-oligomeric structures [11]. Such *cis-trans* isomerization of the Xaa-Pro peptide bonds catalyzed by PPIases is one of the strongest rate-limiting steps of folding mechanism. Both types of immunophilins, FKBPs and CyPs, are active characters in these protein folding steps. 

It is interesting to point out that, despite immunophilins show similar PPIase enzymatic activity, the sequence and structural properties of the FKBP and CyP subfamilies are not equivalent. Nonetheless, immunosuppressant drugs compete with the substrate for binding to the PPIase active site in both types of proteins. Therefore, the PPIase domain of FKBPs (also known as FK1 region) has become almost a synonymous of the drug-binding domain of these immunophilins. 

Not all functions of immunophilins are related to the PPIase enzymatic activity since these proteins are themselves molecular chaperones. Nevertheless, the limits between these two properties are sometimes unclear [12]. For example, to exert effective immunosuppression both FK506 and cyclosporine A form hetero-oligomeric complexes with host cell FKBPs and CyPs. This leads to a gain-of-function property that favours the inhibitory effect on calcineurin. The use of inhibitors lacking immunosuppressive action and/or the assay of genetically modified enzymes exhibiting reduced PPIase enzymatic activity indicates that many of the biological actions of PPIases should possibly occur as consequence of their direct interactions with the prolyl bonds. 

## 3. General Aspects of FKPB51 and FKBP52

The immunosuppressive action of FK506 or cyclosporine A is responsibility of the drug binding to the smallest members of both classical immunophilin subfamilies, i.e. FKBP12, encoded by the *FKBP1A* gene, and cyclophilin A (CyPA), encoded by the *PP1A* gene. As consequence of these specific interactions, the liganded immunophilin (but not the drug or the protein alone) impairs the activity of calcineurin [13], a Ser/Thr-phosphatase also known as PP2B. Thus, the transcription factor NFAT (Nuclear Factor of Activated T-cells) remains phosphorylated in the cytoplasm of lymphocytes, and the production of interleukines and interferon-γ is consequently avoided (see [14] for a comprehensive review for this mechanism). On the other hand, members of the immunophilin family that possess higher molecular weight show more complex protein architecture because they have additional domains to the PPIase domain. One of the best characterized immunophilins is the 52-kDa FK506-binding protein, FKBP52 (gene name *FKBP4*)[15], which is the archetype of the Hsp90-binding subfamily (Figure 1b). In addition to the PPIase domain (also called FKBD1 or FK1 domain), FKBP52 shows three repetitions in tandem of a degenerative sequence of 34 amino acids named tetratricopeptide repeats (TPR), which has the capability to form associations with Hsp90 dimers [16]. 

The Hsp90-binding immunophilin FKBP52 shows a close-related partner, FKBP51 (gene name *FKBP5*), with whom it shares 75% similarity and 60% identity. Due to this high homology, but different conformational features (Figure 1c), both immunophilins usually compete one another for binding and functional properties of client-proteins, although this antagonistic effect shows exceptions in a few numbers of cases and their biological effects become redundant (see afterwards). Both immunophilins possess a short sequence of 7 to 9 amino acids that forms an FK-linker region. It connects the FK1 domain with the FK2 region. The linker sequence of FKBP52 is capped by a TEEED phosphorylation sequence that is substrate of casein kinase-2 (CK2). The resultant phosphorylation at Thr^143^ impairs the FKBP52•Hsp90 interaction [17] and also abrogates the normal regulation seen on steroid receptors by FKBP52. In the case of FKBP51, this loop is capped by FED, a conserved sequence in high molecular weight immunophilins, such that the phosphorylation of the site by CK2 does not occur. Nevertheless, these differences cannot account for the lack of receptor potentiation capability shown by FKBP51, which is most of the times an inhibitor of the biological actions of nuclear receptors.

As it was stated above, it is accepted that the FK1 domains of both immunophilins are the major structural elements responsible for the divergent properties of FKBP51 and FKBP52 on the steroid receptor action [18,19,20,21]. Nevertheless, while the PPIase domain is important in this regard, it appears that the enzymatic activity of prolyl-isomerase is not always essential [20,22]. The immunophilin is often part of a heterocomplex with Hsp90, such that the interaction with the chaperone also influences the FK1 domain of FKBPs [23]. The binding of Hsp90 to the immunophilin enables the FK1 domain to interact with the ligand binding domain of the glucocorticoid receptor (GR), thereby influencing the GR conformation and the steroid binding affinity [24]. Inasmuch as the conformations of the FK1 domains of both FKBPs differ around the PPIase pocket, it is not surprising that this fact causes differential protein interactions with client-factors [25,26].

The structure of the FK2 region (second yellow box in Figure 1b) is similar to that of the FK1 region, but FK2 does not show enzymatic activity of isomerase and cannot recognize immunosuppressive ligands. When the FK2 region of FKBP51 was modified by point mutations, Hsp90 binding took place yet the mutant cannot integrate normally into the receptor heterocomplex [25]. Therefore, it might be possible that the mutation impairs key interactions not only with other members of the receptor heterocomplex, but also with the receptor itself. In contrast to the FK1 domain of FKBP52, the FK1 domain of FKBP51 does not show stimulating activity on steroid receptor activity, but random mutagenesis studies evidenced that two key point mutations in the FK1 domain of FKBP51 confer full receptor potentiation activity to this immunophilin equivalent to that observed for FKBP52 [27]. This suggestive finding is in line with the notion that both proteins may have diverged during evolution by only a very limited number of modified amino acid residues. Inasmuch as these two residues are in the proline-rich loop (see Figure 1c), it may be inferred that this region of FKBP52 is functionally relevant for the regulation of steroid receptor activity. Accordingly, it has recently been postulated that the loop serves as an interaction surface with the ligand binding domain of the receptor [28,29]. 

The TPR domain located at the C-terminal end of the immunophilin confers the capability to interact with the chaperone Hsp90 via the C-terminal sequence of the chaperone, the EEVD motif [16,30]. On the other hand, isothermal titration calorimetry studies performed with FKBP51 and FKBP52 demonstrated that the former immunophilin interacts with Hsp90 dimers with lower affinity (about one third) compared to FKBP52 [31]. Nonetheless, due to the influence of the relative abundance of each FKBPs in each cell type, solely the relative affinity of each immunophilin for Hsp90 cannot permit accurately predict the stoichiometry of the oligomeric complexes formed with the receptors. 

## 4. Immunophilins Play a Key Role in Protein Trafficking

Steroid receptors bind their cognate ligands only if they are assembled with Hsp90 in oligomeric structures [32,33], this Hsp90-based complex being a biological on/off functional switch. This property agrees with the properties of Hsp90, a chaperone that at variance of others that prefer partially unfolded clients, favours substrates that possess a preserved tertiary structure. This is particularly notorious for some members of the steroid receptor family such as GR, mineralocorticoid receptor (MR), progesterone receptor (PR) and androgen receptor (AR) [34]. They are transcription factors with properties of phosphoproteins [35,36,37], and are activated by ligand binding. Steroid receptors are assembled with molecular chaperones and co-chaperones, including Hsp90, Hsp70, Hsp40, p23 and a TPR-domain protein, usually a high molecular weight immunophilin such as FKBP51, FKBP52, CyP40 or PP5. The interaction of TPR proteins with Hsp90 (and Hsp70) is conserved in nature and broadly distributed in both animal and plant kingdoms [38,39,40].

**Figure 2:**
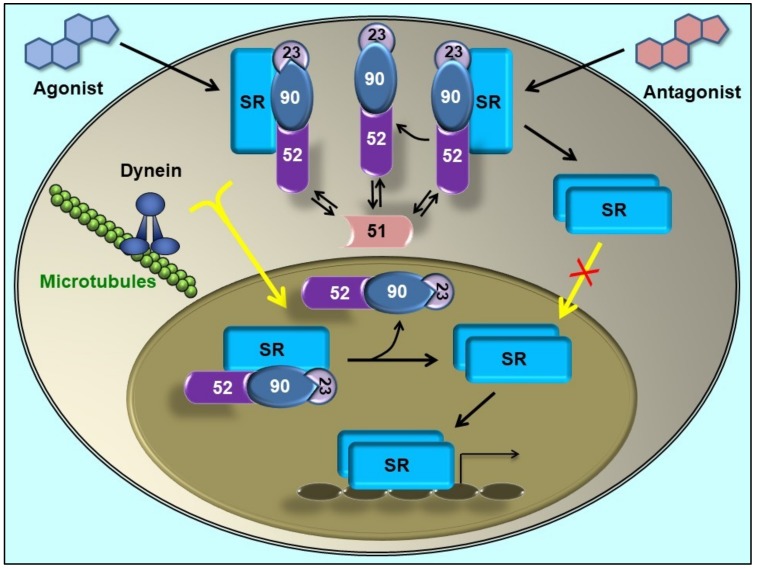
Steroid receptor (SR) retrotransport is affected by immunophilins. The mature form of SR forms complexes with a dimer of Hsp90, one molecule of Hsp70 (not depicted in this figure), the co-chaperone p23, and one TPR-domain immunophilin [51]. FKBP51 (unable to interact with dynein [18]) is associated to the empty SR. When the steroid binds, FKBP51 is exchanged by FKBP52 due to a ligand-induced conformational change of the receptor [52]. In turn, FKBP52 recruits the dynein-dynactin motor complex to its PPIase domain [41]. The complex is rapidly (T_0.5_= 5 min) transported to the nucleus on cytoskeleton tracks. SR ‘*transformation*’ (i.e. the dissociation of the Hsp90-based complex) is a nuclear event [42]. Some antagonists can promote the release of Hsp90 in the cytoplasm, such that SR does not reach the nucleus [50]. Note that regardless of the SR primary localization, they are constantly cycling between the nuclear and cytoplasmic subcellular compartments [45,53], even when the final equilibrium may be displaced to a given cell compartment. When the steroid promotes the full nuclear accumulation of the SR, it still cycles. The disruption of the “transportosome” by any means (Hsp90-disrupting drugs, dynein inhibitors, overexpression of the PPIase or TPR domain, ATP depletion, low temperature, etc.) does not prevent SR movement, but it is one order of magnitude slower (T_0.5_= 45–60 min) than the active mechanism. It is thought that this residual movement represents the diffusion of the complex through the crowded filamentous milieu [54] (Figure adapted from [55],with permission from the publisher).

The discovery that the dynein/dynactin motor complex co-immunoprecipitates with GR [41] and MR [42] via its association to the PPIase domain of FKBP52 modified the classic view for the mechanism of steroid receptor activation. In the absence of steroid, some receptors such as GR, MR or AR (which also interacts with dynein/dynactin, as it was further demonstrated [43]) are primarily cytoplasmic proteins. Upon steroid binding, they rapidly accumulate in the nucleus. Other receptors such as the estrogen receptor (ER) are constitutively nuclear even in the absence of steroid, but they are not statically confined to a cell compartment, but are continuously shuttling between cytoplasm and nucleus [44,45,46,47]. Classically, the driving force for soluble protein movement throughout the cytoplasm was always assumed to occur by simple diffusion, and steroid receptors were not the exception. The classic model for receptor activation posited in the ‘80s proposed that Hsp90 should dissociate from the receptor upon steroid binding to release the transcription factor from the cytoplasmic anchorage sites. When the key role for FKBP52 in the receptor retrograde movement was demonstrated, the classic dogma was replaced by a model where the entire receptor•Hsp90•FKBP52•dynein complex moves throughout the cytoplasm, translocates intact through the nuclear pore, and is finally dissociated in the nucleoplasm [42,48] (Figure 2). A direct corollary of this novel model is the prediction that receptor dimerization cannot be a cytoplasmic event because the association of the receptor with the Hsp90 chaperone complex (a key requirement for receptor retrotransport) blocks the dimerization domain; therefore, it may be predicted that the oligomeric chaperone complex should be released in the nucleoplasm after the translocation through the nuclear pore, which would allow receptor dimerization. This prediction was effectively demonstrated time after for the GR [49] and the MR [50] using different methodologies.

On the other hand, the highly homologous partner FKBP51 does not bind dynein [18]. In line with this fact, it was demonstrated that FKBP51 favours its recruitment to unliganded receptor and is exchanged by FKBP52 when the steroid binds [52,56]. Such dynamic immunophilin exchange has biological relevance due to the differential action exerted by each co-chaperone on the final biological response of the receptor [57,58]. In agreement with the modern model, a very recent study by nuclear magnetic resonance (NMR) spectroscopy analysis [59] evidenced that the FK1 and FK2 domains populate respectively an ensemble of bound and unbound receptor conformation. Also, it was recently suggested that the helix_1-3_ loop in the ligand binding domain of GR is responsible for the regulatory properties of both FKBPs [60]. In a different publication, the conformational transition of the FK1 domain of both immunophilins in their respective association with the GR was analysed [22], and the evidence showed that the interactions in the β_4–5_ loop and the β_2–3a_ strands tend to lock FKBP52 into a conformation that preferentially binds to a high affinity state of the steroid receptor, whereas FKBP51 shows preference for the empty receptor. These structural evidences agree with the biological behaviour of both immunophilins in intact cells. According to the novel model, the competitive balance between dynein-binding immunophilins versus non-dynein-binding TPR proteins associated to the receptor•Hsp90 complex may influence the basal subcellular redistribution of the receptor [42,54]. This could be achieved by regulation of the expression of a given immunophilin or by using drugs able to affect immunophilin function. Accordingly, the overexpression of the TPR domain of the immunophilin or whole FKBP51 protein retains higher amounts of steroid receptor in the cytoplasm [42], whereas drugs able to disrupt the interaction of FKBP51 with the receptor•Hsp90 complex like benztropine restores the expected subcellular localization of the receptor in ex vivo brain slices and primary neurons from mice [61].

Importantly, this Hsp90•FKBP-dependent trafficking machinery has also proved using other models [18,42,62,63,64,65], and has also been demonstrated for a great variety of factors such as the catalytic subunit of telomerase [66], the insect ecdysone receptor [67], or the diphtheria toxin [68], just to mention a few examples. Therefore, the discovery that immunophilins are involved in the “transportosome” molecular machinery has changed substantially the classic molecular model for the mechanism of action of steroid receptors posed heuristically in the literature, but never proved, and helped to explain the cytoplasmic transport of other soluble proteins that share the same chaperone machinery [67,68,69,70,71,72]. It is interesting to point out that the immunophilin-like protein FKBPL/WISP39 shares the same properties as FKBP52 for the regulation of GR retrotransport, including the interaction with dynein motors [73]. It is possible that this type of functional redundancy may also occur with other members of the immunophilin family such as CyP40 and the immunophilin-like protein phosphatase PP5. These two proteins are known components of the Hsp90-based heterocomplex of steroid receptors and their PPIase domains also bind dynein [38].

Regarding the relative abundance of Hsp90-binding immunophilins able to interact with the motor protein in liganded steroid receptor•Hsp90 complexes, it has been shown that while empty GR, MR and PR preferentially associate with FKBP51 over FKBP52 and CyP40 [56,74,75], in the presence of steroid FKBP52 and PP5 are predominantly recruited (along with dynein). However it is CyP40 the dominant immunophilin for ERα•Hsp90 complexes [76], although FKBP52 is also recovered with this receptor. The AR also binds FKBP51 and FKBP52, also being retrotransported by dynein/dynactin motors [43]. Interestingly, the AR shows normal splicing variants that lack the hinge region [77], a short negatively charged segment that lies just C-terminal to the PPIase domain of FKBP52. Due to amino acid charge complementation, this region is the same that has been postulated as an FKBP52 interacting domain with steroid receptors [44,78]. As it may be predicted, the variants of these receptors are unable to interact with dynein, and cells expressing them are insensitive to taxane treatment [77]. Nevertheless, in the case of AR both immunophilins show a peculiar property when they are compared to other steroid receptors, i.e. they are functionally redundant from the transcriptional perspective. 

Because the association of dynein with FKBPs has also been demonstrated in plant systems [38,39], the functional role of this complex seems to be preserved along the evolution. Importantly, the disruption of Hsp90 function is critical to abrogate receptor transport, which is not surprising if we consider that this chaperone is the main scaffold factor of the trafficking molecular machinery. The most relevant extrapolation of this property is the fact that those drugs able to interfere with the chaperone impair the biological action of the client-protein. This is the rational strategy for those ongoing clinical trials that are testing Hsp90 inhibitors for cancer treatment [79,80], particularly due to the broad governing functions shown by the chaperone to regulate most of the hallmark processes proper of malignancies [40] and proteinopathies [81] 

Another important discovery in the immunophilin field related to the endocrine mechanism of action of steroid receptors was the hormone-dependent biological activity of the receptors, which is itself affected by the type of immunophilin present in the complex. New World primates have the characteristic to show plasma parameters of glucocorticoid resistance syndrome with high levels of plasma cortisol, but a normal GR. In 2001 it was demonstrated in squirrel monkeys that FKBP51 decreases both steroid binding capacity and transcriptional activity of the GR, properties that have been directly correlated with the very high levels of expression of endogenous FKBP51 in the cells of these primates [25,82,83,84]. On the other hand, the expression of FKBP52 is significantly lower. These observations indicated that a high level of FKBP51 expression contributes to glucocorticoid resistance. Later, the inhibitory action of FKBP51 on the GR-dependent response was also correlated with the expression of certain polymorphic isoforms of this immunophilin in neurons, this GR resistance being associated to the development of stress-related post-traumatic syndrome and other psychiatric disorders [85]. 

In contrast to FKBP51, it is regarded that FKBP52 enhances the biological response of the GR [86]. This was reported in a yeast reconstituted system where no endogenous immunophilins are expressed and after transfecting the GR, immunophilins and a gene reporter. In our own hands, we have also experienced greater transcriptional activity of GR in FKBP52-KO cells when the immunophilin was reintroduced by transfection, but not in wild type cells that seem to be unaffected by FKBP52 overexpression. This conundrum may be explained if it is reasoned that a basal expression of endogenous immunophilin is enough for the stimulatory effect, which could require sub-stoichiometric amounts of FKBP52. In line with this interpretation, it has also been reported that GR-regulated genes are not significantly affected by the loss of FKBP52 [55] in mice with targeted ablation of the FKBP52 gene, suggesting that the immunophilin may not be an essential global enhancer of GR transcription as it has always been considered. Nevertheless, the positive effect of FKBP52 on GR response in non-expressing cells is unquestionable and, under physiological conditions, it is clear that FKBP52 is required for an appropriate biological response of the receptor upon hormone stimulation.

## 5. Immunophilins in Steroid Receptor-Related Cancer

Even though FKBP51 and FKBP52 are ubiquitously expressed, the variable expression balance between them in different cell types and tissues may regulate the biological functions of the associated receptors. This is particularly important for the cases of hormone-dependent breast and prostate cancers, where ERα and AR are the main targets for anti-hormonal therapy. Interestingly, in breast cancers the level of expression of both immunophilins FKBP52 and CyP40 is strongly up-regulated compared to the normal tissue [87,88,89]. Accordingly, both immunophilins are the main co-chaperones associated to Hsp90 in these complexes. An opposite protein expression ratio is measured in breast cancer cell lines versus normal breast cells, being the Hsp90•FKBP52 complexes more frequent and abundant than Hsp90•CyP40 complexes [87]. Moreover, ERα expression is greatly correlated with higher levels of FKBP52, such that it is currently accepted that the relative loss of CyP40 in the complex compromises ERα biological properties by affecting the immunophilin composition in ERα•Hsp90 heterocomplexes. This fact must impact anti-oestrogen resistance resulting in phenotypic changes in breast cancer. 

FKBP52 is also strongly expressed in prostate cancer cells [90,91]. The immunophilin seems to favour the efficiency of the AR biological actions, a property that is of particular relevance for those cases of therapies based on androgen ablation. In these situations, the plasma levels of androgens are greatly reduced, but they can still generate a response via the AR•Hsp90 complexes [92]. The analysis of needle prostate biopsies in humans revealed that FKBP52 is indeed a useful and reliable biomarker of prostate cancer [91]. Its close-related partner FKBP51 is also overexpressed in this type of cancers and shows the ability to stimulate AR activity [93,94,95], the immunophilin being itself a product of the AR activity [96]. This generates a harmful feedback circuit that is worsened by the fact that FKBP51 impairs the biological activity of the GR. A direct consequence of these properties is that the glucocorticoid-activated GR can attenuate the biological actions of androgen-activated AR genes, suggesting an AR-dependent mechanism where GR can actually function as a tumour suppressor in cases of prostate cancer [97,98]. Accordingly, it has been shown that the mere overexpression of GR is enough to decrease the proliferation rate of prostate cancer cells expressing AR [99], and the activation of the GR by cognate agonists attenuates the expression of androgen-activated AR genes [97], emphasizing the tumour suppressor function of GR on AR. Because FKBP51 is overexpressed in cancer cells, the oncogene activity of AR is enhanced and the tumour suppressor action of GR results inhibited, which contributes to the bad prognosis related to the expression of this immunophilin in prostate cancer [58,100].

Importantly, the macrolide FK506 inhibits androgen-induced cell proliferation [90], an effect abolished by the FKBP51 knock-down, but not by FKBP52 knock-down [101,102]. This implies that no other TPR-domain protein could be an efficient replacement for the function of these immunophilins in these Hsp90 heterocomplexes. Accordingly, the overexpression of FKBP51 favours prostate cancer cell growth and impairs the effectiveness of antiandrogen therapies, for example, treatments with bicalutamide, that is frequently used in patients undergoing androgen ablation therapy [94]. 

Because of the above-described properties of AR with respect to its associated FKBPs, it may be predicted that it is unlikely that FKBP52 is a global and indispensable modulator of the AR. Accordingly, it has been shown that AR functions are not significantly affected by FKBP52 loss in the testes of KO mice [103,104]. As discussed above, this could arise from compensation by FKBP51, which also promotes AR activity and also shows increased recruitment to AR in FKBP52 KO cells, while there is no increase of FKBP51 expression in testes of null mice [102]. Although the PPIase activity is not necessary for enhancing AR activity, the FK1 catalytic pocket appears to be essential in potentiating the AR response to androgens via [105], such that treatments with FK506 reduce AR binding capacity and prevents the androgen-induced proliferation of prostate cancer cells.

There are various studies showing remarkable changes in the expression of both immunophilins, FKBP51 and FKBP52, including cases of oesophageal adenocarcinoma, oral cell squamous carcinomas, and hepatocellular carcinomas. This suggests that both PPIases could be useful prognosis biomarkers for these diseases [106,107,108]. In a recent study, it has been demonstrated that FKBP51 is up-regulated two orders of magnitude in cases of taxol-resistant therapies [109]. In line with these observations, while immunophilin silencing favoured the positive action of paclitaxel treatment in taxol-resistant cells, FKBP51 overexpression favoured the drug resistance. This is a mechanism that involves the AR, not only in prostate cancer cells, but also in ovarian cells. This features are surely associated to those findings where newly identified taxol resistance genes have been reported as FKBP51-regulated, such that silencing of these genes accordingly sensitized cells to taxol [109]. Consequently, it was recommended that taxol should not be used in cases of ovarian cancer where the Protein Kinase B (or AKT)/FKBP51/AR axis is activated [109]. 

The high expression of FKBP51 in cases of colorectal adenocarcinoma tissue have been associated with an immature phenotype of stromal fibroblasts and with the epithelial-to-mesenchymal transition phenotype [110]. This indicates again a possible relation between disease and FKBP51 expression. It is interesting to point out that, since just a certain number of cells of the stroma express the immunophilin, it was proposed a that FKBP51 should play a relevant role in stroma cell subtypes and could be useful as a novel biomarker [111]. FKBP51 was detected by immunohistochemistry of human biopsies in the nuclei of enterocytes of healthy tissue, whereas in colorectal cancer cells the immunophilin was in both nuclei and cytoplasm, the distribution range being variable according to the patient from strong signal to undetectable. Interestingly, liver metastases show a weak or null immunostaining, while the inflammatory fibrous stroma shows a strong signal. However, such variability could also be influenced by the chemotherapy treatment of the patients used for the study rather than due to intrinsic changes of transformation of cells [111]. Based on the trafficking model depicted in Figure 2, the authors of these studies assigned the observed biological effects of these cells to anomalies in the steroid receptor signalling due to the contribution of high level of expression of FKBP51. 

While a proteomic study showed that FKBP52 is highly expressed in hepatocarcinomas [112], the information available for FKBP51 is not prolific. However, in a recent report it was shown that FKBP51 greatly increases its expression upon treatment with a specific micro-RNA (miR-367-3p), whose expression is positively correlated with AR expression in advanced hepatocarcinoma and functions as a metastasis suppressor [113]. This supresses cell proliferation and cell invasion compared to cells with low or negligible expression of the immunofilin. Therefore, it is inferred that there are relatively low levels of expression of FKBP51 (or high FKBP52/FKBP51 ratio) in hepatocarcinoma cells during the early steps of the disease. In advanced states, FKBP51 could be induced following AR increased activity.

## 6. Immunophilins Regulate NF-κB Activity

NF-κB (Nuclear Factor Κ-light-chain-enhancer of activated B cells) is indeed a relatively large family of transcription factors that regulate the expression of genes associated to pleiotropic processes such as the immune response, inflammatory diseases, cell development, cell growth, cancer progression, neuronal synaptic plasticity, memory, cell differentiation, etc. [114]. Members of the family share homologous structure with the retroviral oncoprotein v-Rel, this being the reason why the family is also named NF-κB/Rel [115]. It is known that Rel oncoproteins form homodimers or heterodimers, with the sole exception of Rel B that is only present in heterodimers. 

NF-κB proteins show variable abundance and composition of the heterodimers according to the tissue and cell type. Despite of this variety, the most frequent complex in almost all tissues and cell types is the p50•RelA/p65 heterodimer. p50•RelA/p65 heterodimers are primarily cytoplasmic complexes in resting or unstimulated cells thanks to its association to the IκB inhibitory subunit. Upon cell stimulation (Tumor Necrosis Factors (TNF), lypopolysaccharides (LPS), peroxides, cytokines, etc.), the inhibitor of κB (or IκB) dissociates from the soluble cytosolic dimer allowing the nuclear relocalization of the heterodimer. Such retrotransport of NF-κB is also dependent of the dynein/dynactin motor complex [116]. Like SRs, NF-κB dimers are also subject of a dynamic nuclear-cytoplasmic shuttling [117,118]. Therefore, the IκB/NF-κB complex also undergoes dynamic dissociation and reassociation events. In short, the NF-κB nuclear-cytoplasmic shuttling is quite similar to that described above for steroid receptors, where the inactive cytoplasmic form of these ligand-dependent transcription factors must translocate to the nucleus upon cell stimulation with steroid hormones. In a recent study, it was shown that both immunophilins, FKBP51 and FKBP52, also modulate the nuclear translocation of the p50•RelA/p65 complex and affect the transcriptional activity of NF-κB [69]. Like the case of the GR, FKBP51 also impairs the nuclear translocation rate of p50•RelA/p65 and inhibits the transcriptional activity. This is due to two main reasons—due to the incapacity of FKBP51 to bind dynein/dynactin [18], and because FKBP52 shows in parallel a very potent stimulatory action on NF-κB-dependent transcriptional activity. The main difference with respect to steroid receptors focuses on the biological actions for NF-κB, which are not Hsp90-dependent. Importantly, such Hsp90-independence represents a novel regulatory mechanism for FKBPs. Like the proposed novel regulatory mechanism of action for steroid receptors, the biological action of NF−κB may also be regulated by the expression balance of both immunophilins in different tissues and cell types. Like in the cases of the glucocorticoid biological actions, this interpretation may explain in part the pleiotropic actions of NF-κB in different tissues and cell types for both transcription factors. 

A crucial nuclear mechanism for gene expression is the modification of the chromatin environment of the respective genes. Thus, chromatin factors able to modify chromatin architecture play a key role in this regulation. PPIase proteins participate of this process, Pin1 being one of the first PPIases studied in this regard [7]. Actually, Pin1 is an immunophilin-like protein that possesses a PPIase domain and shows isomerase activity, but this is not regulated by immunosuppressive drugs. Importantly, Pin1 targets RelA/p65 [119]. Because PPIase-induced conformational changes do affect the properties of target proteins, the action of Pin1 on RelA/p65 is obviously reflected in the behaviour of the transcription factor. For example, it facilitates a more efficient nuclear accumulation of the RelA/p65 subunit, and also favours a greater stability of the transcription factor because its ubiquitin-mediated proteolysis is prevented. Pin1 is frequently up-regulated in various cancer cell types [120,121,122] whereas the E3-ubiquitin-ligase of RelA/p65 known as suppressor of cytokine signalling-1 (SOCS-1) [123] is down-regulated or mutated, all of which favours the constitutive activation of NF-κB in those cancers. Similarly, a parallel regulatory mechanism is proposed here for the expression balance of FKBP51 and FKBP52. This is particularly relevant for FKBP52, an immunophilin that possesses an important stimulatory action on NF-κB biological effects in a PPIase-dependent fashion.

In contrast to the strong inhibitory action of FKBP51 on NF-κB signalling in fibroblasts, this immunophilins shows opposite action in melanocytes. Since FKBP51 facilitates so called Ivanhoe the king's knight-IκB kinase (or IKK) complex assembly by physical interaction with IKKα subunits [124], this effect was assigned to the control of NF-κB positive activation as a cofactor of IKK. Due to cell-context-related, differential impairments of NF-κB-regulated gene expression can occur and the strict IKK dependence on FKBP51 might be underlined. Because the prevalent effect of FKBP51 in malignant melanoma is stimulation, it could be a useful target for radiosensitizing strategies [125]. Another interesting example of biological divergent actions is the case of pancreatic cancer. Even though the expression of FKBP51 is indeed high in several types of cancer, its expression in pancreatic cancer is surprisingly down-regulated or directly absent [126,127]. FKBP51 was proposed as a sort of tumour suppressor [128] related to AKT phosphorylation that works on downstream genes of the AKT pathway. Nonetheless, the exact reasons for its opposite behaviour with respect to other types of tumours remain unclear to date.

## 7. AKT/mTOR Signalling Cascade

This pathway is often activated in a constitutive manner in several types of cancer, a reason by which it is an attractive pharmacologic target [129]. Normally, the inactive Ser/Thr-kinase AKT, also known as Protein Kinase B (PKB), is in the cytoplasm, and results associated to the plasma membrane when the cell is activated by growth factors. Phosphatidylinositol-3-kinase (PI3K) is the most significant down-stream signalling activator recruited by tyrosine-kinase receptors. PI3K activity is essential for AKT relocalization to the plasma membrane [130,131]. Conversely, phosphatases like protein-phosphatase 2A (PP2A) or PH-domain leucine-rich repeat protein phosphatase (PHLPP) inactivate the AKT pathway. A major down-stream target of AKT is the kinase mammalian target of rapamycin (mTOR), which exists in two oligomeric forms, mTORC1 and mTORC2. 

FKBP51 regulates AKT phosphorylation status through a scaffolding mechanism, which impacts on the biological response to a variety of antineoplastic compounds [126]. The mTORC1 complex is inhibited by rapamycin (sirolimus) via its binding to FKBP immunophilins [132]. Thus, complexes of this macrolide with FKBP12, but also with FKBP51 or FKBP52 mask the docking sites restricting the access of the kinase to its cognate substrates [133]. Importantly, FKBP51was found as a scaffolding protein able to increase PHLPP•AKT interaction, which favours the PHLPP-mediated dephosphorylation of AKT. Consequently, low levels of FKBP51 expression complexed with rapamycin appear to be enough to prevent the phosphorylation of mTORC1 substrates. This effect is important because FKBP51 is generally overexpressed in most types of cancer cells [72,134]. 

These findings led to study numerous mTORC1 inhibitors with the purpose to treat cancer (e.g., everolimus, temsirolimus, dactolisib, AZD8055, etc. [135,136]. Temsirolimus has recently been approved for the treatment of advanced renal cell carcinoma, and the second generation mTOR inhibitors derived from everolimus has been successfully used along with the aromatase inhibitor exemestane to specifically treat advanced-stage, hormone-receptor-positive and HER2-negative breast cancers in postmenopausal women [137]. More recently, everolimus was also approved for the treatment of neuroendocrine tumours and advanced renal cell carcinoma [138]. 

## 8. The hTERT•Hsp90•FKBP51/FKB52 Complex

Recent studies have demonstrated that some immunophilins are also mitochondrial factors. Among them, it is particularly interesting the case of FKBP51 [72,139,140,141] because it is highly abundant in this organelle (~50% of the cell pool [139]). Nevertheless, FKBP51 lacks a classical mitochondrial localization signal. Interestingly, mutants in the TPR domain of FKBP51 that impair the interaction of the immunophilin with Hsp90 demonstrated that FKBP51 is constitutively localized in the nucleus. This important finding reveals that Hsp90 is responsible for the extra-nuclear localization of the immunophilin. Moreover, it is quite reasonable to postulate that the Hsp90-dependent mechanism for mitochondrial import of proteins [142] is the one used by FKBP51 to be translocated into the organelle. 

It was demonstrated that FKBP51 shows antiapoptotic action and moves from mitochondria to the nucleus upon the onset of stress conditions (deficit of nutrients or high production of reactive oxygen species) [72,139], and during the differentiation of preadipocytes to adipocytes [141]. Interestingly, a shuttling in the opposite direction (from nuclei to mitochondria) was also observed in cells infected with virus or transfected with dsRNA [140]. Note that the first two conditions are frequent situations in cancer tissues, where FKBP51 is often overexpressed [72]. Another typical property of cancer cells is the high telomerase enzymatic activity, which permits their efficient clonal expansion [143]. Telomerase is a ribonucleoprotein that compensates for the loss of telomeric DNA by adding repeated sequences to the chromosome ends. This is achieved by using an intrinsic RNA component as a template for DNA synthesis. Cell generations are counted by telomeres, which constitute a particular molecular device moulded by thousand repeats of a short sequence element located at the extreme of each chromosome [144]. This chromosome-end undergoes the loss of DNA fragments after each cell cycle, a repetitive process that leads to the progressive shortening of telomeres. This phenomenon is assigned to DNA polymerases failure to completely replicate those sequences at the ends of the chromosomes during the S-phase [145]. This mechanism prevents cancer development because the number of cell divisions become obviously limited. When cells lose the control of the cell cycle and consequently become cancerous, they divide more frequently than normal cells, such that their telomeres become shorter after each cycle. This explains why a mechanism for telomere elongation becomes essential for cancer cells to divide indefinitely, to the point that they may reach an immortalization state [146].

While the protein expression and enzymatic activity of telomerase are very low and sometimes absent in most multicellular eukaryotic organisms, the human telomerase reverse transcriptase (hTERT) activity is significantly high in stem cells and germ cells, as well as in specific types of blood cells. Telomerase is always up-regulated in all these cases, which is also a common feature for the vast majority of cancer cells [147]. Notably, hTERT (the reverse transcriptase subunit of telomerase) is a known Hsp90 client-protein responsible for the catalytic enzymatic activity [148,149], whereas hTR (the associated RNA component) is used as the template for synthesis of telomeric sequences. It is remarkable the fact that the Hsp90-based chaperone heterocomplex is required for the proper assembly of hTERT [150] by following an identical folding mechanism to that first elucidated for steroid receptors. Therefore, it is not entirely surprising that both Hsp90-imunophilins, FKBP51 and FKBP52, belong to the hTERT•Hsp90 heterocomplex [66,72]. From the functional perspective, both immunophilins are strong activators of the telomerase enzymatic activity [72], which represents an important observation for cancer cells where both immunophilins are overexpressed and concentrated in the nuclei, as it is expected for cells that undergo several types of stress. hTERT transport to the nucleus uses the same “transportosome” molecular complex [66] already described for steroid receptors. The stress-dependent translocation of chaperones to the nucleus impacts in turn on the amount of immunophilin associated to the enzyme heterocomplex, which at the end favours the enhancing action of the co-chaperones and consequently, cancer cell survival.

Importantly, the enzymatic activity of telomerase is abrogated by the macrolide FK506 or by transfection of FKBPs carrying point mutations in the PPIase domain, but the protein-protein interaction between hTERT and the immunophilin remains unaffected. Consequently, it is entirely reasonable to conclude that the PPIase isomerase activity is not required for the complex assembly, but it is crucial for the enzymatic biological response [72]. This property is quite interesting because transforms immunophilins in novel pharmacologic targets to prevent the clonal expansion of cancer cells simply by impairing the stimulant action over the catalytic subunit of telomerase. In this regard, the recent development of synthetic small molecules able to selectively prevent the PPIase activity without showing immunosuppressive action represents a promising pharmacologic alternative to be explored [61,151]. 

## 9. Role of Immunophilins in Malignancies

Several studies have demonstrated that FKBPs are important for the initiation and progression of various types of cancer, as well as for potential treatments of endocrine-related diseases through perturbation of the steroid receptor signaling cascade (see [106,152,153] for recent reviews). While FKBP51 expression is favored when ligand-activated steroid receptors induce specific transcriptional activity, this immunophilin can also modulate its own expression by a negative feedback loop. Importantly, the overexpression of FKBP51 shows an efficient protective action on cells exposed to several types of stress, whereas its knock-down increases cell sensitive to harmful stimuli and exacerbates cell death. These observations correlate with an enhanced antiapoptotic mechanism mediated by FKBP51. In line with these observations, the expression level of FKBP51 is frequently high in most cancer cell lines and human tumors. Perhaps the triad of the best characterized diseases where FKBP51 is highly expressed are prostate cancer, lymphoma, and melanoma. In prostate cancer and melanoma, the high expression of the immunophilin correlates well with the metastatic potential of these cancer cells. Nonetheless, there are exceptions to this general rule since there are reports showing that the expression of FKBP51 is decreased in specific diseases such as pancreatic tumors. In other cases, the experimental observations showed variable results, such that high expression of FKBP51 correlates with either suppression or promotion of tumor growth, a phenomenon that depends on the type of tumor and its relative microenvironment (see [107,110,154,155,156] for recent studies in this field). Thus, some studies have demonstrated down-regulation of FKBP51 in pancreatic cancer cells [126,127]. Others have reported increased expression in neoplastic melanocytes during the non-invasive (radial) growth phase of cutaneous melanomas with low immunoreactivity. In contrast, it was also evidenced stronger signal in tumor cells of the invasive (vertical) growth phase [125]. High FKBP51 immunoreactivity was also found in all types of metastatic melanoma cases [125]. Interference studies by siRNA evidenced that FKBP51 can prevent the proliferation of colorectal adenocarcinoma, whereas antagonists of the GR impair the effect of transfections of siRNA for FKBP51 on colorectal adenocarcinoma development. Thereby, the suppression of the proliferation via FKBP51 may be due to the suppression of the response mediated by the GR [157]. 

Hsp90 is involved in many malignant phenotypes related to cell invasion and metastasis. Accordingly, the chaperone participates in the activation of Rho (Ras-homologous) proteins and the generation of stress fibers [158], events that exert a direct role in cell migration and invasion processes. A similar involvement of the chaperone has been demonstrated for the activation of the vascular endothelial growth factor-Rho-associated protein kinase (VEGF-ROCK) pathway in cell migration [159]. Not surprisingly, it has recently been reported that FKBP51 is also related to the Rho-GTPase cascades and consequently, to cell motility and cancer cell invasiveness [160]. This is related to the fact that FKBP51 is an interacting partner of deleted in liver ancer-1 and -2 proteins (DLC-1 and -2 proteins), Rho GTPase-activating proteins that are frequently downregulated in various types of cancer. The overexpression of FKBP51 enhances cell motility and cell invasion by up-regulation of RhoA activity and enhanced Rho-ROCK signaling. In contrast, FKBP51-depleted cells show down-regulation of RhoA activity and actin filaments consequently display cortical distribution. Therefore, both cell motility and cell invasion are impaired, a final effect assigned to the role of FKBP51 in cytoskeletal rearrangement and cell migration.

Interestingly, treatments of prostate cancer cells with FK506 relates to the inhibition of the androgen-dependent response mediated by the AR. In turn, this mechanism may be related to a reduced intrinsic steroid binding capacity of AR [93]. When AR-positive human prostate carcinoma LNCaP cell line was compared to PC-3 and DU145 AR-negative prostate cancer cell lines, the inhibitory action of FK506 on cell growth was evidenced only in the AR-positive cells when they were treated with steroid [90]. Therefore, it is possible that the drug may function on the AR-dependent mechanism of cell growth via the associated PPIase protein, in particular FKBP51. Thus, the knock-down of FKBP51 impaired cellular events where this immunophilin promotes AR-dependent transcriptional activity. Interestingly, similar results to those reported for FKBP51 were also observed for the cyclophilin CyP40 [93]. 

High expression of FKBP51 has been found in metastatic melanomas, and the knock-down of the immunophilin was sufficing to highly sensitize cells to ionizing radiation [125]. This effect was assigned to the potential decreased of the anti-apoptotic signalling mediated by NF-κB in response to lower levels of expression of FKBP51. This biological response could be cell- or tissue-specific since a reduced expression of FKBP51 resulted in a low sensitivity to chemotherapeutic agents in breast, lung, and pancreatic cancer cell lines. 

On the other hand, in breast tumors FKBP52 expression is significantly high, and estrogens up-regulate transcriptionally and post-transcriptionally its expression (recently reviewed in [28]). Interestingly, it has been shown that the FKBP52 gene is methylated in the MDA-MB-231 (ER^-/-^) cell line, whereas it is methylated in MCF7 cells (ER^+/+^). This suggests that FKBP52 repression could affect ER expression [161]. Even more importantly, this observation implies that this PPIase protein could be a potential pharmacologic target in cases of breast cancer. 

It has been shown that, in cases of the aggressive non-Hodgkin lymphoma of T/null cell called anaplastic lymphoma kinase-positive/anaplastic large cell lymphoma (ALK^+^-ALCL), the tyrosine kinase enzymatic activity of the Hsp90-binding oncoprotein nucleophosmin-anaplastic lymphoma kinase (NPM-ALK) induces the expression of both immunophilins, FKBP52 and Cyp40, but not that of FKBP51 [162]. It is accepted that CyP40 is the critical immunophilin of the complex because its knock-down decreases the viability of ALK+ ALCL cell lines, an effect that cannot be achieved after knocking down FKBP51 or FKBP52. 

Recently, it was demonstrated that FKBP51 is also naturally overexpressed in glioma cell lines and human glioblastoma samples [163]. Glioblastoma is the most dangerous and aggressive form of brain cancer. Glioblastoma multiforme (a grade IV astrocytic tumor) is the most frequent brain tumor in adults, and shows high rate of mortality [164]. Currently, the curative attempt for glioblastoma is not possible in most patients. The use of the recently developed inhibitor for FKBP51, SaFiT [151], showed promising results by disrupting the biological action of the immunophilin. In other recent study [165], it was reported that NIM811 (MeIle4-cyclosporine), a cyclosporine derivative with high affinity for CyPs uncapable to interfere with T-cell activation because it shows no affinity for calcineurin [166], also induces death in glioblastoma cells, as well as the use of rapamycin, since these cells have high level of activation of the AKT/mTOR pathway [167]. 

As it was outlined before, CyP40 and FKBP52 are greatly expressed in most cases of human breast carcinoma and in most breast cancer cell lines. Such expression is favored by an ER-dependent fashion. Both PPIase proteins associates to the ER in a mutually exclusive manner, and its expression and recruitment to the receptor are increased in breast cancer cells. Although both isoforms ERα and ERβ are the mediators of the effects of estrogen, both receptors have distinct effects. Actually, the biological action of estrogen ligands as risk factors for developing tumors depends primarily on the balance between these two receptors [168], as well as on the high levels of exposure to estrogens during the life time period. In addition to CyP40 and FKBP52, other major player in ER complexes is the immunophilin-like protein FKBPL/WISP39, which shows the capacity to interact preferentially with ERα [169]. FKBPL/WISP39 has been associated with cancer, regulation of tumour growth and angiogenesis, its high expression being positively evaluated for improved patient survival [170], most likely due to stabilization of newly synthesised cyclin-dependent kinase inhibitor, p21.

Estrogens exert their tumorigenic effect via the ERα isoform, whereas the ERβ-dependent effects in breast cancer growth and development is still unclear. Nevertheless, there is experimental evidence that support the notion that a negative dominance of ERβ versus ERα could be responsible of the biological action [171,172]. Thus, it results clear that the ERβ isoform antagonizes the ERα isoform in critical biological responses such as cyclin D1 expression, c-myc repression, or cyclins D1 and A-gene transcription, whereas it increases the expression of the multifunctional cyclin-kinase inhibitors p27^Kip1^ and p21^Cip1^. Therefore, cells become arrested in the G2 phase of the cell cycle. In line with these observations, it has been evidenced that the expression of ERβ is significantly high in normal mammary tissue compared to those cells where the tumour progresses from the pre-invasive to the invasive estate [89]. While the binding of immunophilin ligands weakly prevents the estradiol-induced gene expression, such effect is pronounced in ERβ expressing cells, suggesting that the ERβ isoform is more sensitive to immunophilin inhibitors than the ERα isoform. A similar observation was made when inhibitors of the chaperone Hsp90 such as geldanamycin derivatives and radicicol were tested [89]. Nonetheless, the immunosuppressive primary action of a conventional immunophilin-binding drug such as FK506 or cyclosporine A make these drugs unlikely to be used for other types of therapeutic uses different from immunosuppression (for example, to inhibit ER response), unless novel derivatives devoid of that primary action can be designed and clinically studied in trials. 

Taken all these antecedents together, it may be concluded that immunophilins play various key roles in several types of cancers, which transforms these proteins in attractive therapeutic targets in malignancies. In this regard, a recent study by M.Cox’s group [29] showed that small molecules can be used to inhibit AR biological activity by inhibiting the steroid-dependent dissociation of the AR•Hsp90•FKBP52 heterocomplex. This property results in a lower nuclear accumulation of AR. Interestingly, assays performed in both early and late stage of prostate cancer cells have shown that these compounds can prevent AR-dependent gene expression as well as the proliferation of prostate cancer cells stimulated by androgens [29]. Table 1 summarizes some relevant aspects of FKBP51 and FKBP52 in malignancies.

## 10. Immunophilins and Cell Differentiation 

PPIase proteins are also abundant in the nervous system, their expression level being about 10-50-fold higher than that of the immune system where they were discovered and isolated. Both neuroregenerative and neuroprotective actions are triggered by FK506 in vitro and in animal models. This interesting effect was first assigned to the action of the drug on the low molecular weight immunophilin FKBP12, the effect being via calcineurin/PP2B activation. However, FKBP12 knock-out mice still respond to FK506 treatment and the development of synthetic ligands lacking immunosupressive action confirmed that the Ser/Thr-phosphatase is not involved.

Our laboratory has reported the FKBP52•Hsp90•p23 heterocomplex distributes in perinuclear structures in both undifferentiated neuroblastoma N2a cells and embryonic hippocampal neurons [178]. In a culture medium lacking other trophic factor, including serum, but supplemented with the PPIase ligand FK506, the ring-like structures disassembly and chaperones spread throughout the cytoplasm. Importantly, that original perinuclear area first stained by the chaperones becomes transcriptionally active. Simultaneously, rounded undifferentiated cells acquire a neuronal phenotype that is accompanied not only by the proper induction of neuronal markers such as Tau1, Map proteins, βIII-tubulin, etc., but also the molecular chaperones Hsp90, Hsp70, p23, and FKBP52. Interestingly, FKBP51 remains constant. Our interpretation for this functional subcellular redistribution of the chaperone heterocomplex is that chaperones may be originally repressing the expression of key neuronal genes required for the neurodifferentiation process. Upon cell stimulation with trophic factors, it is entirely possible that the observed chaperone release from the nuclear periphery can favor the early expression of essential factors for the neuronal differentiation. 

In stimulated cells, FKBP52 concentrates in the nascent neurites and arborization bodies, whereas the cochaperone p23 binds to intermediate filaments. Simultaneously, microtubules acquire higher filamentary organization. On the other hand, FKBP51 (whose expression level remains unaffected) occupies those areas where FKBP52 was first present in the perinuclear rings. The importance of the immunophilin FKBP52 in neurodifferentiation is evidenced in cells where the immunophilin is overexpressed: cells differentiate spontaneously even in the absence of FK506, and the addition of the macrolide to the medium induces faster neurite outgrowth, longer neurites and higher number of ramifications, whereas its knock-down strongly delays or abolishes neurodifferentiation. On the other hand, FKBP51 overexpression shows the opposite action, i.e. impairment of differentiation, whereas its knock-down accelerates the differentiation process since the “inhibitor” factor is no longer expressed [178]. Both immunophilins also show converse effects in the reorganization of the cytoskeleton in other systems [189]. Thus, while FKBP51 favours the binding of the microtubule-associated protein Tau with Hsp90 in HeLa cells, it also promotes the stabilization of microtubules, and regulates the phosphorylation status of Tau, a property that is dependent on the PPIase enzymatic activity [190]. On the other hand, FKBP52 promotes microtubule disassembly [191].

In neurons, the section of neurites with a laser beam followed by a reincubation with FK506 led to the full recovery of the neurite phenotype. This was enhanced by overexpression of FKBP52 and impaired by overexpression of FKBP51 [179]. In other words, these experiments suggested that neuroregeneration and neurodifferentiation may follow the same molecular mechanism.

In the nuclear periphery, subdomains are dynamically identified, some of them being repressive and enriched in facultative heterochromatin, while others are permissive for transcription to occur. This architecture is related to the peculiar subcellular distribution of FKBP52 during the early steps of neurodifferentiation. For many years, it was thought that the chromatin associated to the nuclear lamin is not in a permissive state for transcription. Trimethyl-histone H3-Lysine 27 is a common marker for repressed promoters of genes enriched in the nuclear periphery of embryonic stem cells and decreases as cell differentiate. The immunophilin FKBP51 was found associated to lamin and FKBP52 to the trimethyl histone H3 Lysine 27, and the histone moves from periphery to the central areas of the nucleus in the presence of FK506.

Another interesting hint for a role of FKBP52 in neurons was the demonstration of its interaction with the copper-binding metallo-chaperone Atox1 [192]. FKBP52 overexpression stimulates copper efflux, an observation that suggests that the immunophilin may protect neurons against copper toxicity. If this observation is correct, it may be of therapeutic interest since alterations in metal homeostasis have been related to several neurodegenerative diseases such as Parkinson’s disease, Alzheimer’s disease, amyotrophic lateral sclerosis, and also prion diseases [193,194]. Nonetheless, this cannot explain per se the neuroprotective and neuroregenerative properties of the macrolide FK506. 

Immunophilins also play a relevant role during the differentiation of fibroblasts to adipocytes. FKBP51 and FKBP52 modified in opposite manner their respective level of expression during the adipogenesis. While FKBP51 increases during the first hours of cell differentiation, the expression of FKBP52 decreases as adipogenesis progresses [141]. This process first demonstrated in cell lines is also evidenced by Western blot analysis in mice samples of adipose tissue [195]. The mitochondrial localization of FKBP51 in normal cells [139] becomes nuclear upon the onset of stress situations. Thus, FKBP51 translocates rapidly (15–30 min) to the nucleus, where it plays an antiapoptotic role [139]. Similarly, when preadipocytes are induced to differentiate with a cocktail containing insulin, dexamethasone and IBMX (3-isobutyl-1-methylxanthine), FKBP51 also translocates rapidly to the nucleus [141], and cycles back to mitochondria within 48 h post-stimulus. This observation may be related to the fact that adipogenesis is regulated by signaling pathways that coordinately modulate the sequential activation of transcription factors required for cells to differentiate [196]. Studies performed with each differentiation agent led to postulate that IBMX and, to a lesser extent the glucocorticoid, are responsible for that rapid relocalization of FKBP51 to the nucleus [141].

It is interesting to point out that when cells are incubated in the presence of the Hsp90 inhibitor radicicol, the interaction of FKBP51 with Hsp90 is disrupted (as it is expected), and FKBP51 translocates spontaneously from mitochondria to the nucleus [139]. In turn, Hsp90 inhibitors such as geldanamycin or radicicol abrogate preadipocytes differentiation [197,198], not only due to the inhibition of typical client proteins such as GR, MR or peroxisome proliferator-activated receptor gamma (PPARγ), but also due to affecting the mitochondrial-nuclear dynamic shuttling of FKBP51 during the differentiation process. Then, the question is why is a transient concentration of FKBP51 required in the nucleus? Among various possible options, the most reasonable explanation relates to those studies where it was evidenced that during cell differentiation, there is fragmentation of nuclear lamina followed by the loss of lamins A, C, B1, and also emerin at the nuclear rim [199]. It is remarkable that fragmented lamin B not only colocalizes, but also interacts with FKBP51 and protein kinase A-cα (PKA-cα) [141]. Several phosphorylation sites are important in the nuclear lamina disassembly, including those for the cyclin B1-(CCNB1)-CDC2 complex, PKC and PKA [200,201]. Therefore, it is possible that the simultaneous accumulation of FKBP51 and PKA-cα in the nuclear lamina may favour the reorganization of the entire system due to the various phosphorylation events that take place during adipocyte differentiation.

FKBP51 also shows other nuclear functions. Among the most relevant, it associates to PPARγ•Hsp90 heterocomplexes modulating in positive manner the biological actions of this nuclear receptor [202,203,204]. PPARγ behaves as a master regulator for cells to acquire and maintain the adipocyte phenotype. Interestingly, the FKBP51 knock-down performed in preadipocytes facilitates their differentiation to adipocytes, whereas the over-expression of this immunophilin impairs the differentiation of 3T3-L1 preadipocyte cells, an effect that may be related to the fact that FKBP51 restrains the adipogenic potential for both receptors, GR and MR [141]. 

The role of FKBP51 in adipose tissue was also studied in knock-out mice [24]. It was shown that *Fkbp51*-knock out mice do not show an overt phenotype [205,206,207]. Interestingly, these mice possess lower body weight than wild type animals, and their exposure to chronic stress conditions make them to acquire a significant growth of the body weight [207]. This property clearly indicates that adipogenesis cannot be entirely inhibited by the lack of expression of FKBP51. Nevertheless, *Fkbp51* knock-out mice do show reduced lipid accumulation and are resistant to weight gain. In turn, their white adipose tissue is significantly reduced, and also show relatively greater quantities of brown adipose tissue [208]. Analysis of perigonadal and subcutaneous white adipose tissue depots showed high expression of brown adipose tissue genes in *Fkbp51* knock-out mice [208], suggesting that FKBP51 may play a role in the generation of beige fat cells. On the other hand, heterozygous *Fkbp52*-knock-out mice exhibit an increased susceptibility to high fat-diet-induced hyperglycemia. They also show hyperinsulinemia, a fact that correlates well with reduced insulin clearance. These KO mice also show high frequency of hepatic steatosis and glucocorticoid resistance [195]. As for *Fkbp51-Fkbp52* doubled knock-out, it is not compatible with life since embryonic lethality occur in all the cases [24]. This suggests the possibility that both immunophilins possess some physiologic redundancies, which must be uncover by tissue-specific conditional double knock-out. 

Other system where immunophilins have been related to cell differentiation has recently been reported for myoblasts [183]. FKBP51 is involved with this process because it forms heterocomplexes with the cyclin-dependent kinase Cdk4, a kinase whose enzymatic activity is modulated by Hsp90 heterocomplexes [209,210]. Thus, the involvement of this kinase in Cdk4•Hsp90•FKBP51 heterocomplexes prevents the formation of cyclin Cdk4•D1 complexes. AS a consequence, cell differentiation becomes greatly impaired [211]. The second molecular mechanism demonstrated in the same study involves the PPIase activity of FKBP51, i.e. a cis/trans isomerization of the Thr^172^-Pro^173^ peptide bond of Cdk4, which in turn inhibits the phosphorylation of Thr^172^, a key requirement for Cdk4 activation. Accordingly, muscle regeneration is delayed in FKBP51-KO mice [183]. Interestingly FKBP52 is not related with this specific differentiation process despite of showing the capability to sequester Cdk4 in complexes with Hsp90. It should be noted that the study demonstrates that FKBP52 is inactive to promote the cis/trans isomerization of Cdk4 and to induce Thr phosphorylation.

## 11. Stress-Related Neurologic Disorders

As it was outlined above, Hsp90-binding immunophilins form oligomeric complexes with steroid receptors. It is particularly important the biological regulation of the GR by FKBP51 in the nervous system. Like in most tissues, FKBP51 is also strongly up-regulated by glucocorticoid stimulation of neuronal cells. As a direct consequence of such neuroendocrine response, different types of stress also trigger an equivalent effect since the plasma levels of this corticosteroid are greatly stimulated in these situations to protect neurons from a harmful condition. In both cases, glucocorticotherapies and stress experiences generate an ultrashort negative feedback loop whereby stimulation of the GR induces FKBP51 expression. Because it represses GR activity [212], this is traduced in an amplification of the stress response if the stimulation is prolonged, which influences several aspects of neuronal function and the tissue synaptic plasticity [213,214].

Genetic variants of the *fkbp5* gene encoding for FKBP51 protein have been consistently associated in humans with psychiatric disorders related to post-traumatic events [20,85,215,216]. One of the most studied single nucleotide polymorphism of the *fkbp5* gene is a variant named rs1360780, which is able to regulate gene expression in neurons and the hypothalamic-pituitary-adrenal axis activity, but other polymorphic variants such as rs4713916, rs1360780 and rs3800737 are also able to modulate the recovery from psychosocial stresses. In these cases, homozygous individuals show an anomalous normalization of the stress-induced cortisol secretion [217,218] and a male-specific effects of the variant rs3800374 to respond to acute psychosocial stress was also found in healthy individuals [219]. The studies revealed that these gene modifications are also associated with personality traits [220] and the functional reactivity of specific brain regions [221,222,223,224,225]. Altogether, this phenomenon reveals a key role of genetic variants of the *fkbp5* gene to assimilate and modulate stress and adversities throughout life, ultimately being one causative reason to develop psychiatric disorders [226], and lead to postulate FKBP51 as an attractive pharmacologic target for these problems. Experimentation in this regard showed promissory initial results after using small molecule antagonists of FKBP51 in murine models. Stress coping was improved, and anxiety was reduced [151,217,227]. Nonetheless, a number of issues still deserve to be taken into account at this point to proceed properly with more ambitious therapeutic strategies to target humans, including the specific expression pattern of the immunophilin in different tissues and the fact that FKBP51 also participates in the regulation of various and important cellular events under normal conditions.

## 12. Concluding Remarks

The immunophilin family comprises relatively “novel” factors whose biological functions have been emerging during the last two decades, although most of the roles in the cell are still to be discovered. Actually, there is poor or almost non-existing information about the biological functions of several members of the family. Unquestionably, FKBP51 and FKBP52 are among the most studied members, and even so, they have generated to date more questions than answers. Since they were first characterized as novel members of the Hsp90-based heterocomplex, relevant advances have been achieved, from the converse capability to facilitate the retrotransport of soluble factors to their involvement in neutrophism. Nonetheless, the conundrum related to most relevant aspects of the biology of these proteins and the regulation of these events is still unsolved. This is particularly true when these proteins are thought as potential therapeutic targets for the effective treatment of those specific medical conditions where it is known they are involved. This pharmacologic aspect is relevant from the perspective that, noteworthy, immunophilins are also an important part of normal mechanisms that regulate physiological parameters in healthy individuals.

The recent development of synthetic compounds able to specifically target FKBP51 without affecting the PPIase activity of its close-related partner FKBP52 [151], is a promising advance in the field. Actually, the combined action with other drugs such as anti-depressant agents has been currently assayed for potential treatments of psychiatric disorders [61,227,228,229]. Probing physiological pathways in response to specific inhibitors of PPIases will become increasingly important during the following years. Until recent times, most of the known actions of immunophilins had been solely related to their chaperone function (i.e. protein-protein interactions) rather than to its isomerase activity. The roles that have been evidenced for the PPIase enzymatic activity on the regulation of AR [90], Cdk4 [183], NF-κB [69] or hTERT [72] may add a new incentive to test existing and eventual new drugs able to interfere with such enzymatic activity in those pathways where these factors are involved. It should also be considered that some members of the immunophilin family show null or negligible PPIase enzymatic activity (the so-called immunophilin-like proteins), although they can be activated or inhibited by other means such as biochemical stimuli or protein-protein interactions (most of them, still to be discovered).

Far from being ideal, the current state of the art for therapeutic applications still requires more exhaustive studies to better understand in-depth the mechanistic aspects of immunophilins, including the demonstration of putative unknown associations with other factors and the subsequent regulation of their corresponding physiologic pathways. 

## Figures and Tables

**Table 1:** Effects of FKBP51 and FKBP52.

	FKBP51	FKBP52	Ref.
Malignancies			
Breast	↑	↑	[173,174]
Prostate	↑	↑	[90,96]
Melanoma	↑	N	[125,175]
Pancreas	↓	N	[126,175]
Oral squamous cell carcinoma	↑	(n.d.)	[107]
Hepatocarcinoma	↓	↑	[112,113]
Colorectal carcinoma	↑	↑	[110,176]
Lymphoma	↑	↑	[162,177]
Nervous System			
Neurodifferentiation	↓	↑	[178]
Neuroregeneration	↓	↑	[179]
Astrocytoma	↑	↑	[180,181,182]
Myoblast differentiation	↑^(*)^	↓	[183]
Adipogenesis	↑	↓	[141]
Steroid receptors			
GR	↓	↑	[18,86]
MR	↓	N	[56,184]
PR	↓	↑	[185,186]
AR	↑	↑	[93,95,187]
NF-κB signaling			
Melanoma cells	↑	↑	[124,175]
Kidney fibroblasts/Placenta cells	↓	↑	[69,188]
mTOR signaling	↑	↑	[131]

↑: stimulation; ↓: inhibition; N: no significant variations; n.d.: no data; ^(*)^: early events. GR: glucocorticoid receptor; MR: mineralocorticoid receptor; PR: progesterone receptor; AR: androgen receptor; NF-κB: nuclear factor κ light chain enhancer of activated B cells; mTOR: mammalian target of rapamycin.
